# Polymorphism of interleukin-17 and its relation to mineral density of bones in perimenopausal women

**DOI:** 10.1186/s40001-014-0069-1

**Published:** 2014-12-16

**Authors:** Dariusz Boroń, Seremak-Mrozikiewicz Agnieszka, Kotrych Daniel, Bogacz Anna, Kamiński Adam

**Affiliations:** Department of Histology and Embryology, Medical University of Silesia, Jordana 19, 41-808 Zabrze, Poland; Division of Perinatology and Women’s Diseases, Poznan University of Medical Sciences, 33 Polna Street, 60-535 Poznan, Poland; Department of Orthopedics and Traumatology, Pomeranian Medical University, Unii Lubelskiej 1 Street, 71-252 Szczecin, Poland; Department of Pharmacology and Phytochemistry, Institute of Natural Fibres and Medicinal Plants, 27 Libelta Street, 61-707 Poznan, Poland; Laboratory of Experimental Pharmacogenetics, Department of Clinical Pharmacy and Biopharmacy, University of Medical Sciences, 14 Marii Magdaleny Street, 61-861 Poznan, Poland; Department of Stem Cells and Regenerative Medicine, Institute of Natural Fibres and Medicinal Plants, 27 Libelta Street, 61-707 Poznan, Poland

**Keywords:** Polymorphism, *IL-17* gene, Osteoporosis, Women, Age

## Abstract

**Background:**

Recognition of different genetic variants underlying osteoporosis would make it possible to introduce individual, symptomatic treatment as well as early prophylaxis of osteoporosis.

The aim of the study was to evaluate the frequency of the rs2275913 (−197G > A) polymorphism of the *IL-17* gene and assess the relation of this polymorphism with the clinical parameters of the osseous turnover and degree of the postmenopausal osteoporosis.

**Methods:**

The study included 800 women of postmenopausal (505) and reproductive (295) ages throughout the Wielkopolska region in Poland. The postmenopausal group included women with osteoporosis and osteopenia, and those who were healthy. Women at reproductive age were healthy. The frequency of the tested gene polymorphism was evaluated in the group where bone mineral density (BMD) was marked and in the control group.

**Results:**

The results obtained showed that the T-score in the female population with osteopenia was remarkably lower in women showing the GG genotype of -197G > A polymorphism of *IL-17* gene compared to patients with heterozygous GA genotype. It has been shown that the BMD value for L2–L4 YA in the evaluated female population with osteoporosis is significantly higher in women with the GA genotype of -197G > A polymorphism of *IL-17* gene compared to women with the GG genotype (76.32% versus 59.93%, *P* <0.05). It has also been noted that the BMD value for L2 to L4 AM in patients with the GG genotype was lower than in women with the AA genotype (69.73% versus 80.88%, *P* <0.05).

**Conclusions:**

It is suggested that the -197G > A polymorphism of the IL-17 gene may be considered as a genetic factor of postmenopausal osteoporosis. This polymorphism can influence the bone mineral density and T-score value in young women and postmenopausal women.

## Background

Interleukin 17 (IL-17) is a proinflammatory cytokine. The gene IL-17 is localized at the short arm of chromosome 6 in position 6p12, coding the 155-length protein product of amino acids. Interleukin 17A was the first cytokine of the IL-17 family to be discovered [1]. Interleukin 17E is produced mainly by Th2 cells, whereas IL-17A and IL-17 F are produced by different types of cells, including T, NK or neutrophils. Moreover, IL-17A shows its highest expression in the Th17 cells, for which it appears as an identification feature.

For some years now, IL-17 and cytokines involved in Th17 lymphocyte production, including IL-23 and IL-21, have been believed to play a significant role in the pathogenesis of rheumatoid arthritis. Today it is known that IL-17 produces not only Th17 lymphocytes but also other types of cells, including macrophages, neutrophils and mast cells [2]. Interleukin 17 shows pro-destructive properties, stimulating also the epithelial, endothelial and fibroblastic cells to produce proinflammatory cytokines and connective tissue that degrades metalloproteinases.

Some later studies have shown that IL-17 is localized in the upper part of the inflammatory cascade and stimulates fibroblasts, synovial membrane cells and macrophages to produce more proinflammatory cytokines. Moreover, IL-17 initiated the loss of proteoglycans and, in chondrocytes, stimulates increases in the expression of enzymes responsible for collagen degradation. Metalloproteinases produced in such processes are regulated by the IL-17 in the presence of the cytokines. It is thought that the IL-17 stimulates osteoblasts to synthesize prostaglandin E_2_ and to express the gene of receptor activator of NF-κB (RANK), which induces osteoclastogenesis. In this way IL-17, is jointly responsible for bone resorption, as with rheumatoid arthritis. More extensive understanding of the mechanism of the effect of IL-17 upon bone and cartilage cells could help introduce new methods of treatment of skeletal system diseases.

Th17 cells have a major role in autoimmune diseases thorough their involvement in development and differentiation of osteoblasts, and when enhanced by IL-23, they secrete, among other things, IL-17A and IL-17 F [3,4]. During the inflammatory process in rheumatoid arthritis, the axis IL-23-IL-17 appears much more important than the loop IL-12–IFN-γ, while the interaction between IL-17 and IL-23 plays an important role not only during the initial phase of inflammation, but also throughout the destructive phase marked by osteoclastogenesis [5,6]. In rheumatoid arthritis IL-23 concentrations correlate to articular fluid IL-17 concentration and serum IL-17 and TNF-α concentrations [7,8].

Interleukin 17 stimulates, among other things, the production of chemokines, other proinflammatory cytokines (for example, IL-6 or TNF-α), proangiogenic factors and cytokines (for example, IL-8), and cartilage degrading metalloproteinases as well as important cytokines promoting osteoclastogenesis that results in bone destruction. In humans differentiation of auxiliary T lymphocytes into Th17 cells depends importantly on IL-6, whereas preserving a constant pool of cells, accomplished through proliferation, is supported by IL-23 [9].

Erosion of the osseous tissue occurs through two routes: from the marrow cavity side, effecting in periarticular osteoporosis and formation of geodes, and from the joint cavity, leading, as a result, to bone erosion. The major reason for bone resorption is the excess of cytokines, which promote the osteoclastogenesis that is induced through RANKL, including IL-1β, TNF-α, IL-6, IL-7 or IL-17, and this process is accompanied by a deficiency of cytokines hampering such process (IFN-γ, IFN-α, IL-4) [10]. IL-17, however, not only enhances the production of osteoclasts, initiated by RANKL, but is also capable of inducing such processes independently [11].

Recent studies have shown that localized at the top of the inflammatory cytokine cascade, IL-17 stimulates fibroblasts, synoviocytes and macrophages to produce more proinflammatory cytokines. Moreover, IL-17 initiates a remarkable loss of proteoglycans and stimulates chondrocytes to produce enzymes that intensify collagen breakdown. Results suggest that IL-17 stimulates osteoblasts to synthesize prostaglandin E2 and to express RANK, effecting osteoclastogenesis induction. IL-17 is probably responsible for enhanced resorption of the osseous tissue. A detailed recognition of such mechanisms is likely to allow for some new therapeutic methods [12,13].

Therefore this study aimed to define the role of relation of the evaluated gene to the process of postmenopausal osteoporosis and bone density, as well as other individual and clinical parameters. The objective was to assess frequency of the rs2275913 (−197G > A) polymorphism of the *IL-17A* gene coding interleukin 17 among postmenopausal women. The subject of analysis will be the relation of the evaluated genetic variant to the stage of osseous changes, as well as relation to the osseous turnover parameters and assessment of the role of the tested genetic polymorphism in etiopathogenesis of osteoporosis.

## Methods

### Patients

The study comprised a group of unrelated Caucasian women inhabiting the region of Wielkopolska. Investigations included 800 women of postmenopausal age (505) and reproductive age (295). The postmenopausal group included 314 women with osteoporosis, 110 with osteopenia and 81 healthy individuals. Densitometric measurements were performed to define the bone mineral density (BMD) as well as T-score, Z-score, and the mean bone mineral density index as compared to the mean value for young adult (YA) women and mean bone mineral density as compared to the mean value for a given age (AM, age matched). Additionally, body weight and height were measured to calculate the body mass index (BMI).

A detailed history of each patient was taken to gain information on the diseases developed, medication prescribed, age of first and last menstruation, number of deliveries, and birth weight, as well as smoking habits.

Those women in whom menopause occurred at least a year ago; who did not receive therapies possibly influencing the bone mass, including selective estrogen receptor modulators – SERM, calcitonin, biphosphonates, heparin, steroids, thyroid hormones, antiepileptic drugs, GnRH analogues; and who underwent no hormonal replacement therapy (HRT) were qualified for genetic tests. Patients who had undergone bilateral ovariectomy, as well as those suffering from endocrine and metabolic disorders, hematological disease, neoplastic conditions, renal disorders, autoimmunologic diseases, or connective tissue diseases, as the above could possibly influence the bony mass, were excluded from the study. Additionally, a group of Caucasian women at reproductive age were examined (mean age: 27.5 ± 4.7).

The study was planned according to statement of Human and Animal Rights. It has been approved by the local Bioethical Committee in Poznan (no. 1415/03 (158/06)).

Bone mineral density (BMD) was measured at the lumbar vertebrae, from vertebra L2 through L4, employing dual energy X-ray absorptiometry (DXA). Densitometric tests were performed with the use of a LUNAR DPX 100 unit (by Lunar Corporation, Madison, WI, USA). BMD scores were expressed as g/cm^2^ and presented as T-score and Z-score indices, referring to mean BMD values for a given age group. BMD scoring between one standard deviation from the mean age referring to the peak bone mass, measured by DXA method, was considered normal (T score from +1 to −1).

Tests comprised the influence of the evaluated polymorphism upon T-score, Z-score, L2–L4 AM, L2–L4 YA, L2–L4 BMD, BMI as well as other clinical parameters.

### Analysis of rs2275913 polymorphism of gene *IL-17A* by real-time PCR

Genetic analysis was performed in the Laboratory of Experimental Pharmacogenetics, at the Department of Clinical Pharmacy and Biopharmacy, Poznan University of Medical Sciences, Poland. DNA was isolated from peripheral blood leukocytes using a QIAamp DNA Blood Mini Kit (Qiagen, USA). Analysis of the -197G > A polymorphism of the *IL-17A* gene was performed with the real-time PCR method with the use of LightCycler™ 480 system. Genotypes were determined using HybProbe probes (TiBMolbiol, Poland), which hybridize on the PCR product and emit a fluorescent signal (FRET- fluorescence resonance energy transfer). Genotyping of the rs2275913 (−197G > A) polymorphism was based on melting curve analysis. LightSNiP set with phials containing proper concentration of starters and probes specific for the amplified fragment, was used to determine the rs2275913 (−197G > A) polymorphism of the *IL-17A* gene. Preparation of the LightSNiP set used for real-time PCR was according to the manufacturers’ instructions. PCR cycling reactions consisted of initial denaturation at 95°C (5 min), and 40 cycles with denaturation (15 s at 95°C), annealing (15 s, 60°C), and extension (15 s at 72°C). Each 96-well plate contained a mixture of case and negative control DNA samples.

### Statistical analysis

Genotype and allele frequencies were calculated for the -197G > A polymorphism and subsequently tested for Hardy–Weinberg equilibrium with the Chi-square test. Cross tables were constructed in order to compare genotypes and allele frequencies between the study population and controls. Odds ratios (OR) and the 95% confidence interval (CI) were calculated. The statistical significance of difference between control and experimental groups was assessed by SPSS 17.0 software using one-way ANOVA test (SPSS Inc.). The values of *P* <0.05 were considered as a statistically significant difference.

## Results

Analysis of the frequency of the GG, GA and AA genotypes of the -197G > A polymorphism of the *IL-17* gene showed comparable results in groups with osteoporosis and without osteoporosis (osteopenic patients and healthy postmenopausal women) (Table 1). No statistically significant differences were noted. In both groups, the heterozygous GA genotype was the most frequently observed.

**Table 1:** **The frequency of genotypes of the -** ***197G > A IL-17*** **polymorphism in women with osteoporosis and without osteoporosis (with osteopenia and healthy)**

**Genotype IL-17**	**Women with osteoporosis**		**Women without osteoporosis**		**OR**	**95% CI**	***P***
	**Observed value n (%)**	**Expected value (%)**	**Observed value n (%)**	**Expected value (%)**			
GG	120 (39.2)	39.2	57 (36.1)	35.0	1.25	0.83 to 1.90	0.15
GA	143 (46.7)	46.8	73 (46.2)	48.3	1.02	0.68 to 1.02	0.49
AA	43 (14.1)	14.0	28 (17.7)	16.7	0.76	0.43 to 1.33	0.18
Total	306 (100)	100	158 (100)	100	-	-	-

In women with osteopenia, the frequency of homozygous GG and AA genotypes and the heterozygous GA variant of the -197G > A polymorphism was comparable to healthy postmenopausal women. No statistically significant differences between the groups were noted (Table 2). In both groups, the heterozygous GA genotype was the most frequently observed.

**Table 2:** **The frequency of genotypes of the -** ***197G > A IL-17*** **polymorphism in women with osteopenia and healthy after menopause**

**Genotype IL-17**	**Women with osteopenia**		**Women without osteoporosis**		**OR**	**95% CI**	***P***
	**Observed value n (%)**	**Expected value (%)**	**Observed value n (%)**	**Expected value (%)**			
GG	35 (35.0)	34.8	22 (37.9)	35.4	0.88	0.43 to 1.82	0.42
GA	48 (48.0)	48.4	25 (43.1)	48.2	1.22	0.60 to 2.47	0.33
AA	17 (17.0)	16.8	11 (19.0)	16.4	0.87	0.35 to 2.25	0.46
Total	100 (100)	100	58 (100)	100	-	-	-

Additionally, analysis of frequency of the GG, GA and AA genotypes -197G > A polymorphism of the *IL-17* gene showed comparable results between the study groups including women with osteopenia and osteoporosis (GG = 35%, GA = 48%, AA = 17%) and healthy women of reproductive age (GG = 37.9%, GA = 43.1%, AA = 19%). In both groups, the heterozygous GA genotype was the most frequent (Table 3).

**Table 3:** **The frequency of genotypes of the -** ***197G > A IL-17*** **polymorphism in women with osteoporosis and osteopenia or healthy and of reproductive age**

**Genotype IL-17**	**Women with osteoporosis and osteopenia**		**Women without osteoporosis**		**OR**	**95% CI**	***P***
	**Observed value n (%)**	**Expected value (%)**	**Observed value n (%)**	**Expected value (%)**			
GG	155 (38.2)	38.1	98 (34.6)	35.4	1.17	0.84 to 1.62	0.19
GA	191 (47.0)	47.2	141 (49.8)	48.2	0.89	0.65 to 1.22	0.26
AA	60 (14.8)	14.7	44 (15.5)	16.4	0.94	0.61 to 1.47	0.43
Total	406 (100)	100	283 (100)	100	-	-	-

Moreover, results presented in Tables 4 and 5 showed that the T-score values were significantly lower in women with the GG genotype of the -197G > A polymorphism of the gene *IL-17* compared to patients with the heterozygous GA genotype. As illustrated in Table 6, the BMD value for the L2 to L4 YA in the female population with osteoporosis was significantly higher in women with the GA genotype compared to women with the GG genotype (76.32% versus 59.93%, *P* <0.05). The findings also showed that the BMD value for the L2 to the L4 AM in patients with the GG genotype was lower than in women with the AA genotype (69.73% versus 80.88%, *p* <0.05).

**Table 4:** **Comparison of clinical parameters analyzed and particular genotypes of the -197G > A** ***IL-17*** **polymorphism in healthy women after menopause**

**Parameter/genotype**	**GG**	**GA**	**AA**
T-score	−0.19 ± 0.49	−0.01 ± 0.72	0.32 ± 0.47
Z-score	0.60 ± 0.93	0.63 ± 0.87	0.70 ± 0.72
Body mass (kg)	70.33 ± 9.80	67.07 ± 9.81	67.13 ± 8.56
Height (cm)	163.04 ± 5.66	163.58 ± 6.02	163.97 ± 5.92
Body mass index (kg/m^2^)	25.07 ± 4.07	25.73 ± 3.80	25.10 ± 4.40
Age	52.03 ± 7.60	50.32 ± 8.02	54.68 ± 7.90
Birth weight	3552.83 ± 517.35	3506.79 ± 440.31	3708.44 ± 501.71
Reproductive years	36.63 ± 5.06	36.04 ± 4.72	35.36 ± 4.77
Age of menarche	12.78 ± 1.68	13.92 ± 1.69	13.83 ± 1.88
Last menstrual period age	50.17 ± 4.58	52.16 ± 4.04	51.03 ± 5.03
Number of pregnancies	2.10 ± 1.06	2.06 ± 1.50	1.87 ± 1.26
Years after menopause	7.45 ± 6.06	7.58 ± 5.47	7.90 ± 6.15
BMD L2–L4 (g/cm^2^)	1.14 ± 0.09	1.26 ± 0.15	1.24 ± 0.11
BMD L2–L4 YA (%)	93.45 ± 8.57	99.52 ± 8.06	106.24 ± 7.52
BMD L2–L4 AM (%)	100.02 ± 9.12	106.30 ± 10.02	109.56 ± 9.52

**Table 5:** **Comparison of clinical parameters analyzed and particular genotypes of the -197G > A** ***IL-17*** **polymorphism in women with osteopenia**

**Parameter/genotype**	**GG**	**GA**	**AA**
T-score*	**−2.03** ± 0.31	**−1.68** ± 0.50	−1.80 ± 0.41
Z-score	−0.90 ± 0.70	−0.70 ± 0.71	−0.88 ± 0.66
Body mass (kg)	63.35 ± 9.73	65.02 ± 9.04	67.13 ± 8.52
Height (cm)	163.15 ± 5.17	160.38 ± 4.73	162.02 ± 5.71
Body mass index (kg/m^2^)	25.01 ± 3.66	23.60 ± 3.92	24.18 ± 4.15
Age	56.15 ± 8.80	50.73 ± 7.50	52.64 ± 8.06
Birth weight	3206.52 ± 523.21	3178.24 ± 477.32	3403.06 ± 501.76
Reproductive years	35.36 ± 5.09	35.03 ± 4.73	38.46 ± 4.36
Age of menarche	14.13 ± 1.42	12.83 ± 2.11	13.0 ± 1.82
Last menstrual period age	51.50 ± 4.76	49.02 ± 4.32	50.07 ± 1.53
Number of pregnancies	1.89 ± 1.37	1.92 ± 1.37	1.87 ± 1.53
Years after menopause	7.04 ± 6.44	7.58 ± 6.02	7.63 ± 5.74
BMD L2–L4 (g/cm^2^)	0.95 ± 0.09	1.01 ± 0.15	0.98 ± 0.12
BMD L2–L4 YA (%)	77.84 ± 6.15	86.06 ± 6.74	80.52 ± 5.13
BMD L2–L4 AM (%)	92.44 ± 6.82	90.07 ± 5.70	88.36 ± 6.24

*P < 0.05 – significance of bold entries was observed by post hoc analysis.

**Table 6:** **Comparison of clinical parameters analyzed and particular genotypes of the -197G > A** ***IL-17*** **polymorphism in women with osteoporosis**

**Parameter/genotype**	**GG**	**GA**	**AA**
T-score	−2.93 ± 0.73	−3.30 ± 0.67	−2.97 ± 0.75
Z-score	−1.50 ± 0.66	−1.62 ± 0.50	−1.40 ± 0.72
Body mass (kg)	64.11 ± 8.13	60.07 ± 9.22	59.68 ± 8.47
Height (cm)	163.61 ± 4.89	160.77 ± 4.90	157.82 ± 5.13
Body mass index (kg/m^2^)	25.92 ± 3.62	23.30 ± 4.11	22.61 ± 3.73
Age	60.07 ± 7.90	50.32 ± 8.18	52.13 ± 8.36
Birth weight	3192.68 ± 493.06	3062.23 ± 443.24	2878.24 ± 506.06
Reproductive years	37.28 ± 4.88	35.62 ± 5.39	34.02 ± 4.93
Age of menarche	13.68 ± 1.52	13.59 ± 1.84	12.87 ± 1.97
Last menstrual period age*	47.6 ± 4.82	50.78 ± 5.00	50.32 ± 4.36
Number of pregnancies	1.95 ± 1.09	1.93 ± 1.14	1.99 ± 1.31
Years after menopause*	10.31 ± 5.78	12.37 ± 6.33	12.85 ± 6.04
BMD L2–L4 (g/cm^2^)	0.84 ± 0.06	0.084 ± 0.04	0.082 ± 0.06
BMD L2–L4 YA (%)*	**59.93** ± 6.02	**76.32** ± 6.90	70.23 ± 6.93
BMD L2–L4 AM (%)*	**69.73** ± 6.82	74.56 ± 6.14	**80.88** ± 7.33

*P < 0.05 – significance of bold entries was observed by post hoc analysis.

## Discussion

The present study showed no significant changes related to the impact of the examined *IL-17* polymorphism on the development of osteoporosis. However, a tendency of this cytokine to influence the inhibition of osteoporosis in individuals with the homozygous AA genotype was observed. Hence, it is suggested that the role of IL-17 in bone economy may be manifested as a negative effect. Interleukin 17 is a cytokine associated with some proinflammatory properties, and it is produced by the T cell group, known as the Th17 cells [14-16]. Th17 cells were first identified in animal models of autoimmune diseases [17]. Although Th17 cells have been well characterized in animal models, their presence in humans is currently recognized. The recent study attempted an evaluation of the potential role of IL-17 as a mediator of bone remodeling, including osteoclastogenesis.

Some centers have focused on the potential role of IL-17 in joint damages. Koshy et al. [18] investigated the ability of IL-17 to induce collagen release from the cartilage. It was shown that the cytokine stimulated the release of proteoglycans and type II collagen from the bovine nasal cartilage, depending on the dose. Such information indicates that IL-17 may act as the mediator of the cartilage collagen breakdown. Another center investigated the influence of exogenous IL-17 on production and release of metalloproteinase-1 through synoviocytes isolated from the synovial membrane [19]. The exogenic addition of IL-17 effected a fivefold increase in MMP-1 production by the synoviocyte culture. The same experiment performed with the synovial membrane specimens instead of the synoviocyte culture, showed that the exogenic addition of IL-17 had no remarkable influence upon MMP-1 production, which emphasized the complex cooperation of several types of cells within the synovial membrane. Sato et al. [6] evaluated the role of the Th17 cells in osteoclastogenesis in mice arthritis. In such a model, the loss of osseous tissue was associated with excessive bone resorption by osteoclasts; in the presence of Th17 cells, however, the osteoclasts were recreated. To solve whether Th17 cells themselves or their products, including IL-17, had a role in osteoclastogenesis, they introduced Th17 cells from mice with no IL-17 to the breeding system. Remarkable inhibition of osteoclastogenesis was observed, which pointed to the necessary role of IL-17 in the osteoclastogenic processes. Nevertheless, IL-17 promoted osteoclasts in the co-breeding system, which indicated that this cytokine did not affect directly the osteoclast precursors but rather cells that enhance osteoclastogenesis, that is, osteoblasts. The exogenic addition of the IL-17 induced RANKL expression on the osteoblast surface, in this way facilitating osteoclastogenesis. The potential role of the Th17 cells in the pathogenesis of autoimmunologic rheumatoid arthritis has also been investigated [20]. Arthritic joints actively transcribed IL-17, which was not the case in non-arthritic ones. Mice showing an IL-17 deficiency were resistant to autoimmunologic arthritis, whereas interferon γ deficiency resulted in the disease progression.

It has been known for some years that IL-17 and cytokines involved in the production of Th17 lymphocytes, among other IL-23 and IL-21, have an important role in the pathogenesis of rheumatoid arthritis. Today it is known that interleukin 17 is produced not only by the Th17 lymphocytes but also other cell types, including macrophages, neutrophils and mast cells [2]. Interleukin 17 shows strong pro-destructive properties. It stimulates the epithelial, endothelial and fibroblastic cells to release proinflammatory cytokines and connective tissue degrading metalloproteinases [21,22]. Mice in which the IL-17 was neutralized or in which the expression was stopped by means of genetic engineering showed a milder course of bone and joint disease. Therefore, clinical studies are being performed to use antibodies neutralizing this cytokine [2].

Patients suffering from joint and bone disorders show enhanced osteoclastogenesis and activity of osteoclasts along with impaired bone remodeling [23]. It is known that osseous tissue erosion follows two routes: from the marrow cavity, effecting in osteoporosis, and from the joint cavity, leading to bone erosion. In normal conditions, RANKL occurs in osteoblasts, whereas under pathological conditions, the sources are other cells. The major reason for bone resorption is the excess of cytokines (including IL-17), promoting osteoclastogenesis through RANKL induction, accompanied by deficiency of cytokines hampering such process [10]. On the other hand, IL-17 not only enhances production of osteoclasts, as initiated by RANKL, but is also capable of inducing such process independently [11].

## Conclusions

Our results suggest that the -197G > A polymorphism of the IL-17 gene may be considered as a genetic factor of postmenopausal osteoporosis. This polymorphism can have the influence on bone mineral density and T-score value in young women and postmenopausal women.

## Abbreviations

AM: age matched
BMD: bone mineral density
BMI: body mass index
CI: confidence interval
DXA: dual energy X-ray absorptiometry
HRT: hormonal replacement therapy
IFN-γ: interferon gamma
IL: interleukin
MMP-1: matrix metalloproteinase-1
NF-κB: nuclear factor kappa-light-chain-enhancer of activated B cells
OR: odds ratios
PCR: polymerase chain reaction
RANK: receptor activator of nuclear factor κ B
RANKL: receptor activator for nuclear factor κ B ligand
SERM: selective estrogen receptor modulators
TNF-α: tumor necrosis factor alpha
YA: young adults
